# Network medicines^™^

**DOI:** 10.1186/s12967-023-04657-8

**Published:** 2023-10-31

**Authors:** Joyce Hu

**Affiliations:** Sonata Therapeutics, Watertown, MA USA

I am delighted to announce the launch of a new section dedicated to *Network Medicines*^™^. This section will highlight biology-based, integrative approaches aimed at understanding the topology of complex disorders and the dynamic interplay of their various components, identifying cascades of causes and effects, and deciphering crosstalk among cells to suggest novel and more effective therapeutic approaches.

Network medicines^TM^ include interactions of systems components from molecular to cellular communications. Molecular communication can be defined as measurable exchanges among linked artificial or biological entities through molecules that digitally encode messages. This principle can extend to bioproducts and to cellular systems, where computational tools are used to define patterns and make predictions about the effect of perturbations over complex systems.

Network medicines^TM^ follows the premise that the solution to complex diseases does not necessarily need to be complex [1]. To quote Albert-László Barabási who originally coined the concept of “network medicine” [2], “*biological systems, similarly to social and technological systems, contain many components that are connected in complicated relationships but are organized by simple principles*.”

We strongly believe that limitations in the efficacy of standard therapeutics is due not only to the incomplete understanding of their root cause but also to the approximate and minimalistic approaches commonly employed to treat multifaceted disorders. These approaches are designed to exploit single mechanisms of action aimed against partially relevant determinants of the disease. Integration of various modalities into a unified product can nowadays be achieved by cutting edge synthetic biology, cellular reprogramming and creative structural engineering methods that go far beyond the paradigm of standard therapies, as recently discussed in the context of adoptive cell therapy [3].

Network medicines^TM^ represent an aspect of Translational Medicine that relies on the bidirectional and interactive development of comprehensive disease atlases accounting for disease heterogeneity and adopts non-linear mathematics to study the regulation of biological networks [4]. The purpose is to build disease atlases with the accuracy matching that of global positioning systems. This can be done by providing a comprehensive framework of information collected from experimental and clinical data that can identify methods to resolve disease in the most efficient pathway. An interactive training and validation process powered by machine learning algorithms may expedite the discovery and understanding of multifactorial mechanisms that underly chronic self-sustaining conditions such as cancer, fibrosis, degenerative disorders, and neurologic disorders.

Cancer serves as an outstanding example of a complex disorder that is determined by the germline background of the patient; the evolving somatic mutations and genetics of cancer cells; environmental, anamnestic, social, and behavioral factors; and co-morbidities with their respective related treatments [5]. Similarly, the fibrotic progression of non-alcoholic fatty liver to cirrhosis and cancer depend on the complex interplay between distinct cellular components in the liver microenvironment. These diseases can only be addressed by the deconvolution of multifactorial causality [6] and the influence played by other multifaceted disorders such as obesity and insulin resistance [7].

The holistic approach that we advocate relates to networks at various levels including biophysical interactions among subcellular components, intracellular signaling, metabolic pathways, or higher levels of crosstalk involving multicellular networks such as the distinct immune landscapes that can determine the prognosis and responsiveness to cancer therapy [8].

Several sections of the *Journal of Translational Medicine* address aspects related to network medicines^TM^. We believe that a new section fully dedicated to this integrative approach will complement other sections by evaluating submissions that go beyond descriptive observations to provide compelling recommendations of disruptive therapeutic candidates.
